# Downregulation of S100A4 expression by RNA interference suppresses cell growth and invasion in human colorectal cancer cells

**DOI:** 10.3892/or.2011.1598

**Published:** 2011-12-20

**Authors:** LIYONG HUANG, YE XU, GUOXIANG CAI, ZUQING GUAN, SANJUN CAI

**Affiliations:** 1 Department of Colorectal Surgery, Fudan University Shanghai Cancer Center, Shanghai 200032, P.R. China; 2 Department of Oncology, Shanghai Medical College, Fudan University, Shanghai 200032, P.R. China

**Keywords:** colorectal cancer, S100A4, RNAi, invasion

## Abstract

S100A4 protein, a member of the S100 superfamily of calcium-binding proteins, is frequently observed in various types of human cancers, including colorectal cancer (CRC). Our previous investigations have demonstrated that the overexpression of S100A4 is associated with lymph node metastasis and poor prognosis in CRC; however, its biological roles in CRC remain unclear. In the present study, we compared the expression of S100A4 at the mRNA and protein levels in six CRC cell lines, and found that the expression levels roughly coincided with their invasiveness. Using RNA interference, we suppressed S100A4 expression in SW620 CRC cells with highly invasive potential and S100A4 high expression. The specific knockdown of S100A4 strongly suppressed cell growth, migration and invasion activities. Furthermore, employing metastasis-related gene mRNA microarrays, we found four genes to be significantly dysregulated (more than 2-fold) after downregulation of S100A4, including three downregulated genes (MMP9, MMP10 and CDH11) and one upregulated gene (TIMP4). Our present results indicate that S100A4 may positively regulate tumor cell proliferation, invasion and metastasis associated with multiple molecules. Thus, the inhibition of S100A4 might be a potentially novel approach to treatment for CRC.

## Introduction

Colorectal cancer (CRC) is the third most prevalent human cancer worldwide, with about 1 million new cases annually. It is estimated that more than 500 thousand people will die from CRC every year (1). Despite advances in both earlier diagnosis through screening and better treatment modalities which have decreased the mortality in the past decades, there is still no effective method for increasing the overall survival rate of affected patients (2). In order to improve the horrible prognosis, identification of new targets of molecular therapy, especially those that are indicative of proliferation and invasiveness, is urgently needed.

Previously, we identified that the overexpression of S100A4 is associated with lymph node metastasis and poor prognosis in CRC revealed by proteome analysis (3). S100A4 belongs to the S100 superfamily, a multi-gene family of Ca^2+^-binding proteins of the EF-hand type (4). S100A4-mediated calcium signaling is involved in a variety of cellular processes, such as immune response, differentiation, cytoskeleton dynamics, cell growth, and cell adhesion and motility (5–7). However, the biological function of S100A4 involved in the development and/or progression of cancers still remains poorly understood.

In recent years, RNA interference has been employed as a powerful strategy for investigating the functions of molecules that promote the initiation, progression and metastasis of malignancies by downregulating the expression of targeted molecules in diverse human cancers (8–12). In this study, we applied small interfering RNA (siRNA) technique to specifically suppress S100A4 expression in the CRC cell line SW620, which expresses a high level of S100A4 and possesses high invasiveness, to investigate the phenotypic changes including the proliferative, invasive and metastatic activities. Furthermore, we explored the significantly dysregulated genes after S100A4 silencing using metastasis-related gene mRNA microarray analysis.

## Materials and methods

### CRC cell lines and cell culture

The six CRC cell lines Caco-2, HCT116, SW480, Colo 205, Lovo and SW620 used in this study were obtained from the American Type Culture Collection (ATCC, Rockville, MD, USA). All cells were cultured in the corresponding medium (recommended by the suppliers) supplemented with 10% fetal bovine serum and 1% penicillin-streptomycin, and were maintained in a 37°C incubator with 5% CO_2_.

### Real-time PCR

Total-RNA was extracted using the RNeasy mini kit (Qiagen, Valencia, CA, USA), and the concentration was detected by a biological spectrophotometer. Real-time PCR analysis was performed according to the manufacturer’s instructions (the Quant SYBR-Green PCR kit, Tiangen Bioteck, Beijing, China). β-actin was applied as an internal control. The primers for β-actin (205 bp) were 5′-TGACGTGGACATCCGCAAAG-3′ (sense) and 5′-CTGGAAGGTGGACAGCGAGG-3′ (antisense). The primers for S100A4 (185 bp) were 5′-GCCCTGGATGTGATGGTGT-3′ (sense) and 5′-TCGTTGTCCCTGTTGCTGTC-3′ (antisense). Each assay was performed in triplicate, and the average was calculated. For relative quantification, 2^−ΔΔCt^ was calculated and used as an indication of the relative expression level.

### Western blotting

A total of 1×10^7^ cells were collected. The cells were lysed and the protein concentrations were measured using a BCA Protein Assay Reagent kit (Pierce, Rockford, USA). A 20 μg aliquot of the protein was subjected to 10% SDS-polyacrylamide gel electrophoresis (PAGE), and then transferred to a polyvinylidene difluoride (PVDF) membrane (Bio-Rad, Hercules, CA, USA). After being blocked by incubation overnight in PBST containing 5% dry nonfat milk, the PVDF membrane was incubated with rabbit polyclonal antibody against S100A4 (1:1,000 dilution; Abcam, Cambridge, UK) for 2 h, and then incubated with a horseradish-peroxidase-conjugated secondary antibody (1:100 dilution; Proteintech, Chicago, IL, USA) for 1 h. Immunoreactive bands were visualized using an ECL detection system (Amersham, Arlington Heights, IL, USA) and quantitated by densitometry using an LAS-3000 imager. β-actin was detected simultaneously as a loading control (anti-β-actin, 1:1,000 dilution; Kangchen, Beijing, China).

### siRNA transfection

The SW620 cells were divided into three groups, namely siS100A4, siControl and mock control. The sequence used to target S100A4 was sense, 5′-GCUGAGCA AGUUCAAUAAATT-3′ and antisense, 5′-UUUAUUGAAC UUGCUCAGCTT-3′. An unrelated oligonucleotide (siControl) recognizing an irrelevant transcript was used as a negative control, and mock control was only transfected with Oligofectamine reagent. These siRNAs were purchased from the company GenePharma (Shanghai, China). Transfection of siRNA, siControl and mock control was performed by the Oligofectamine kit (Invitrogen, Carlsbad, CA, USA) according to the manufacturer’s instructions. Briefly, SW620 cells were plated at 50% confluency with L-15 medium (Gibco, Grand Island, NY, USA) without antibiotics. Before transfection, culture medium was changed to Opti-MEM Reduced Serum Medium (Gibco), and then siRNA and non-sense siRNA were transfected into the cells at the concentration of 100 nM. Oligofectamine reagent alone (0 nM) was applied to the cells as a mock control. After 5 h, the medium was replaced with L-15 medium containing serum. The effectiveness of gene silencing was determined by real-time PCR and Western blotting.

### Cell proliferation assay

Proliferation was assessed by the Cell Counting kit-8 (CCK-8; Dojin, Tokyo, Japan). Briefly, cells of mock control, siControl and siS100A4 were incubated at a density of 1×10^5^/well in 96-well culture plates (Costar, Cambridge, MA, USA) at 37°C for 7 days, and every 24 h each well of the cultured cells was incubated with 10 μl of Cell Counting kit-8 solution for 1 h at 37°C. The absorbance at 450 nm was measured with a spectrophotometer.

### Invasion and migration assay

The cell invasive potential of the three groups of tumor cells was performed using Transwell filters (Costar). Briefly, 32 h after RNA interference, the filters coated with Matrigel (BD Biosciences, Bedford, MA, USA) in the upper compartment were applied with 100 μl of 1×10^5^ cells seeded in serum-free medium, and the lower compartment was filled with culture medium supplemented with 10% fetal bovine serum. The plates were incubated in a humidified 5% CO_2_ incubator at 37°C for 36 h, the cells on the top membrane surface were gently scraped with a cotton swab, and the migrated cells on the bottom surface were fixed with methanol and counted after staining with toluidine blue. The cell migration assay was performed in a similar mode, except that the cells were seeded into the uncoated filter and incubated for 24 h.

### Tumor metastasis PCR array

Total-RNA from siS100A4 and siControl was extracted with the RNeasy mini kit (Qiagen) and further purified by the use of a RNeasy MinElute™ Cleanup kit (Qiagen). The RT^2^ First Strand kit (Qiagen) was employed to produce a cDNA library for the total-RNA extracted. Following the manufacturer’s protocol, the cDNA was then processed to perform the Human Tumor Metastasis RT^2^ Profiler™ PCR Array (Qiagen, Mississauga, ON, Canada) containing 84 genes known to be related to tumor metastasis, five housekeeping genes used for a genomic DNA control, and three positive controls to ensure high quality data normalization across samples. The results were analyzed by SA Biosciences software. For relative quantification, 2^−ΔΔCt^ was calculated and used as an indication of the relative expression level.

### Statistical analysis

All experiments were performed in triplicate. Results are expressed as means ± standard deviation (SD). A two-tailed Student’s t-test was performed to analyze the statistical significance of differences between experimental groups using the SPSS 11.5 software statistical package. A P-value <0.05 was considered to denote statistical significance.

## Results

### S100A4 expression analysis in six human colorectal cancer cell lines

**Figure 1:**
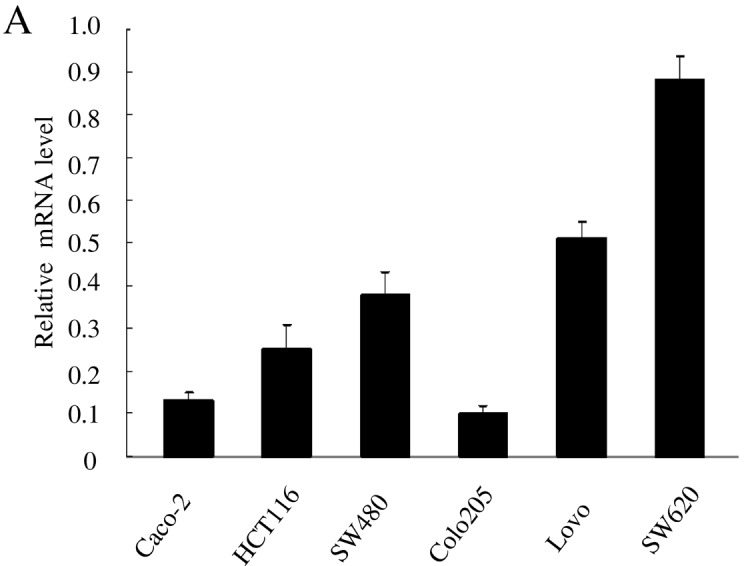

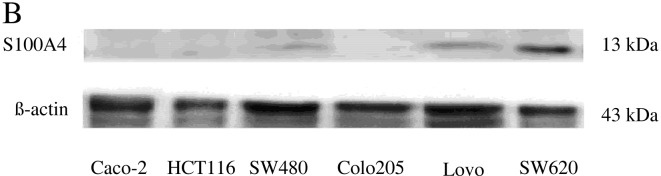
S100A4 expressions in six colorectal cancer cell lines. (A) Real-time PCR analysis of S100A4 expressions. Data represent the mean ± standard deviation (SD) of three independent experiments. (B) Western blotting of S100A4 expressions. The S100A4 expression in the SW620 cell line was highest at the mRNA and protein level, and the expression levels of the six cell lines roughly coincided with their invasive potential.

We first examined the expression of S100A4 by quantitative real-time PCR in six colorectal cancer cell lines. The result showed that S100A4 expression was the highest in the SW620 cell line followed by Lovo cells, and low in other cell lines, which roughly coincided with their invasiveness (Fig. 1A). Western blotting also showed the consistent expression of S100A4 in the six cell lines (Fig. 1B). In addition, many studies have suggested that the SW620 cell line possesses highly invasive potential among CRC cell lines. Taken together, we selected the SW620 cell line for further characterization.

### Downregulation of S100A4 expression by siRNA silencing in SW620 cells

**Figure 2:**
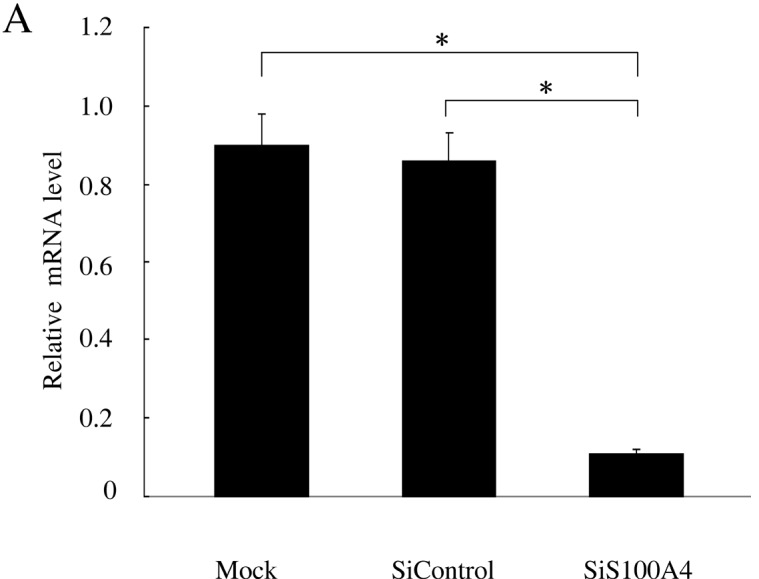

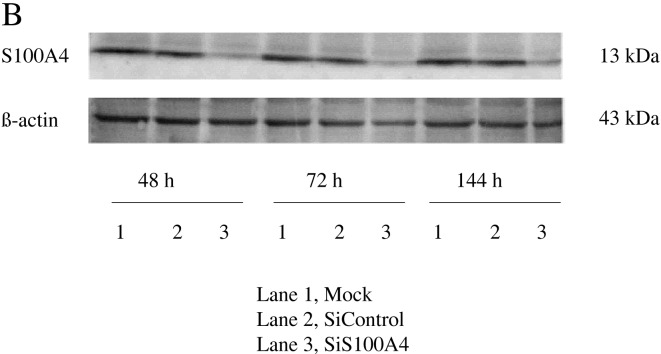
S100A4 gene knockdown by siRNA transfection in SW620 cells. (A) By real-time PCR, S100A4 mRNA expression was significantly suppressed at 36 h post transfection of S100A4 siRNA into SW620 cells compared with mock and siControl of SW620 cells. Data represent the mean ± standard deviation (SD) of three independent experiments. ^*^Specific comparison between the siS100A4 group and mock control, siControl group (P<0.05). (B) By Western blotting, the S100A4 protein expression was decreased in SW620 cells at 48 h post-transfection of S100A4 siRNA and remained at low levels at 144 h (6 day) post-transfection. Lane 1, mock control transfected SW620 cells; lane 2, siControl transfected SW620 cells; lane 3, siRNA transfected SW620 cells.

The effect of transfection on the silencing of the S100A4 gene expression was evaluated using both real-time PCR and Western blotting (Fig. 2). After 36 h following transfection, real-time PCR analysis showed that the mRNA expression levels of S100A4 in mock control and siControl of SW620 cells were 0.9±0.08 and 0.86±0.07 respectively, and there was no statistical difference between them (P>0.05). The relative expression of S100A4 was 0.11±0.01 in siS100A4-transfected SW620 cells, which was significantly decreased compared with those in mock control and siControl (P<0.05) (Fig. 2A). After 48 h following siS100A4 transfection in the SW620 cell lines, Western blot analysis also revealed that the S100A4 protein expression was significantly reduced compared with those in the mock control and the siControl, and the reduction persisted for 6 days (Fig. 2B). The data indicate that RNAi effectively suppresses S100A4 expression in SW620 colorectal cancer cells.

### Effect of S100A4 knockdown on cell proliferation

**Figure 3:**
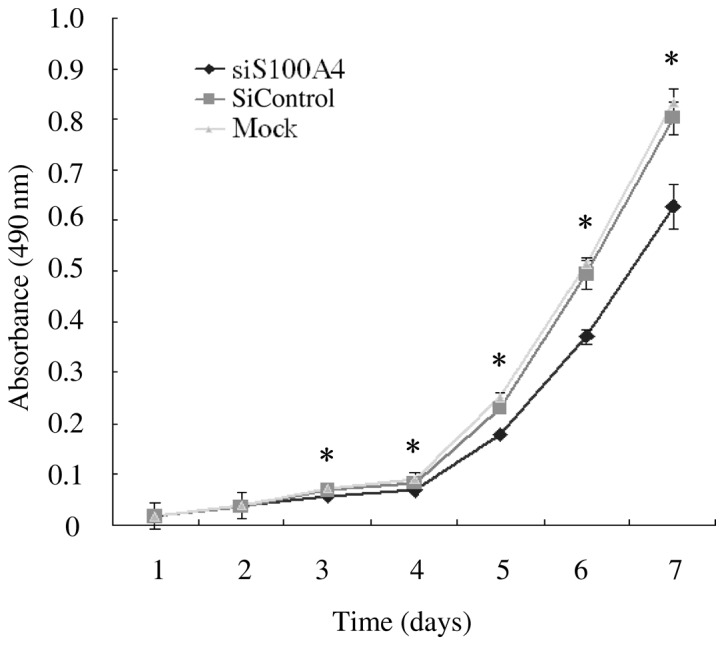
S100A4 siRNA inhibited the proliferation of SW620 cells *in vitro*. Cell proliferation of SW620 cells transfected with mock control, siControl and siS100A4 was analyzed by the Cell Counting kit-8 assay. Cells were seeded in 96-well culture plates (1×10^5^ cells/well). Cell proliferation was determined daily for 7 days in all. The absorbance was read at 490 nm by a spectrophotometer microplate reader. Data represent the mean ± standard deviation (SD) of six independent experiments. ^*^Specific comparison between the siS100A4 group and the mock control, siControl group at Days 3, 4, 5, 6 and 7 of the time course (P<0.05).

To investigate the effect of S100A4 knockdown on functional alteration, growth of cells was detected by a cell proliferation assay. SW620 cells were transfected with siS100A4, siControl, and mock control, and cell growth was assessed daily for 7 days in all. The growth rate in siS100A4 transfected cells was significantly slower than those in siControl transfected cells, and mock-transfected cells (P<0.05 at Days 3, 4, 5, 6 and 7) (Fig. 3). This result indicates that S100A4 mediates cell proliferation in SW620 cells and its suppression leads to inhibition of cell growth *in vitro*.

### Migration and invasion assay

**Figure 4:**
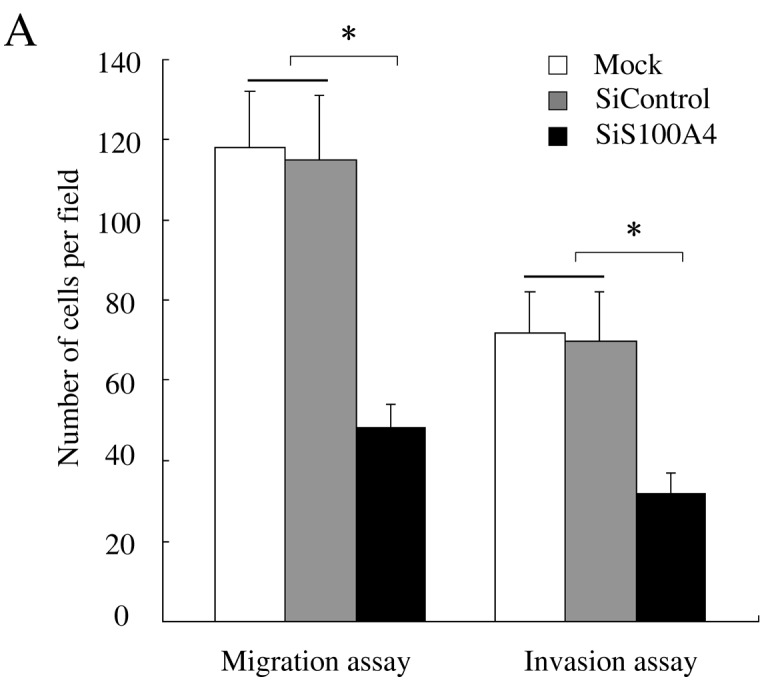

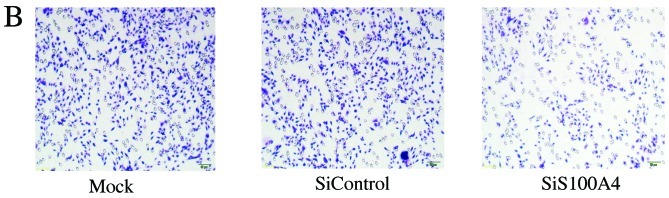
Downregulation of S100A4 by RNAi attenuated migration and invasion activity of SW620 cells. (A) Statistical plots of the number of cells that invaded the Transwell membranes of the haptotactic migration assay and Matrigel invasion assay. Data represent the mean ± standard deviation (SD) of three independent experiments. ^*^Specific comparison between siS100A4 group and mock control, siControl group (P<0.05). (B) The polycarbonate filters were stained with toluidine blue and viewed with a light microscope (magnification, ×100).

We further studied the effect of S100A4 suppression on the migration and invasion of SW620 by mobility assays. The cell migration assay showed that the number of cells that had moved to the bottom chamber was much smaller in the siS100A4-transfected cells than in the siControl and mock-transfected cells (P<0.01) (Fig. 4A). The result of the Matrigel invasion assay also revealed that the number of invaded cells transfected with S100A4 siRNA significantly decreased in comparison with those in the siControl and mock-transfected cells (P<0.05) (Fig. 4A). Fewer siS100A4-transfected cells than siControl and mock-transfected cells were observed when the polycarbonate filters were stained with toluidine blue (Fig. 4B). Our data suggested that gene silencing by S100A4-siRNA can inhibit the invasive potential of SW620.

### Expression profiles after knockdown of S100A4

To further study the possible mechanisms by which S100A4 modulated the growth, invasion and metastasis potential of SW620 cells, we purified total-RNA from the SW620-siS100A4 and SW620-siControl and used PCR array analysis to profile the expression of tumor metastasis-related genes in response to the knockdown of S100A4 expression in SW620 cells. A careful analysis of the results showed that four genes were markedly dysregulated (>2-fold) after S100A4 silencing in SW620 cells, including three downregulated genes (MMP10, MMP9 and CDH11) and one upregulated gene (TIMP4). Based on the attributes and molecular events involved in the metastasis-related procession, the encoded proteins of these dysregulated genes could be categorized as extracellular matrix proteins (MMP9, MMP10, TIMP4) and cell adhesion genes (CDH11) (Table I).

## Discussion

S100A4, also known as 18A2/mts1, CAPL, PEL-98, 42A, p9Ka, and metastasin, belongs to the S100 superfamily of calcium-binding proteins (4). It is located in a 2.05 Mbp segment of the genomic DNA of chromosome 1q21 region (13). The molecule occurs as non-covalently bound homodimers that can interact with an array of target proteins in a calcium-dependent manner (14).

Recently, many studies have shown that S100A4 is an important factor relevant to progression and prognosis in various human cancers, such as thyroid tumors (15), breast (16), pancreatic (17,18), lung (19), gastric (20) and colorectal cancers (21–24). Our previous study found that the overexpression of S100A4 existed more frequently in colorectal cancer patients with advanced stage, lymph node metastasis and was associated with poor prognosis (3). However, the functional significance of S100A4 in colorectal cancer is still not clear. Understanding its functional roles and molecular mechanisms involved in the tumorigenesis and progression of cancers might provide us the opportunity for the early diagnosis and development of novel management for malignancies.

In this study, we first examined the expression of S100A4 in six colorectal cancer cell lines with various invasive potentials at the mRNA level by real-time PCR and at the protein level by Western blotting. The level of S100A4 expression among the six colorectal cancer cell lines roughly coincided with their invasiveness, which was consistent with our previous conclusion that increased expression of S100A4 correlated with increased invasiveness in tumor tissues (3). To demonstrate the role of S100A4 in tumor development and progression, we silenced the expression of S100A4 using RNAi in the colorectal cancer cell line SW620 with highly invasive potential and S100A4 high expression. In order to validate the effect of siRNA targeting on the S100A4 gene, we measured the expression of S100A4 at both the mRNA and protein levels. The results demonstrated that the RNAi against S100A4 can downregulate the expression of S100A4 expression effectively at both the mRNA and protein levels in SW620 cells after transfection. The CCK-8 assay was used to detect the change of cell proliferation in a time-dependent manner daily for a total of 7 days. The result confirmed that the proliferation in siS100A4 cells was reduced significantly compared with those in the siControl and mock control (P<0.05). In addition, assessment of the invasive potential, after transfection with S100A4-siRNA, demonstrated that the rate of cell migration was significantly reduced compared with those in siControl and mock control, suggesting that S100A4 may be an important contributor to the invasion of tumor cells and the expression level of S100A4 can influence the metastatic behavior of SW620 cells.

Tumor metastasis is a complicated process, with a variety of dysregulated molecules playing a significant role (25,26). We used metastasis-related gene mRNA microarrays to analyze the dynamic change of genes before and after S100A4 silencing, which may provide a helpful insight into the potential mechanism of tumor growth and metastasis inhibition due to S100A4 silencing. As a result, we identified that four metastasis-related genes were significantly dysregulated (more than 2-fold) after S100A4 silencing, including three downregulated genes [matrix metalloproteinase 9 (MMP9), MMP10, and cadherin 11 (CDH11)] and one upregulated gene [tissue inhibitors of metalloproteinase 4 (TIMP4)] (Table I), suggesting that S100A4 functions in association with a series of important genes involved in tumor growth and metastasis, instead of the alteration of a single molecule.

MMPs belong to a family of zinc- and calcium-dependent endopeptidases which intrinsically are responsible for the degradation of the components of the extracellular matrix (27). In particular, MMP9 has been identified as a significant molecule contributing to tumor cell metastasis (28). In a human prostate cell line, the expression of MMP9 was significantly reduced after S100A4 suppression by RNA interference (29), which was in agreement with our study. Another MMP, MMP10 was also notably reduced after S100A4 silencing. It has been demonstrated that MMP10 may serve as a diagnostic and prognostic marker in patients with gastric cancer and those with oral cancer, suggesting an important role of MMP10 in cancer progression (30,31). Tissue inhibitors of metalloproteinases (TIMPs) may play an important role in the metastatic process of cancer cells by metalloproteinase inhibition and in other pathways independent of MMP inhibition (32). The expression of TIMP4 was revealed to be a protective factor for prostate cancer progression (33). The mechanisms by which the decreased expression of MMP9 and MMP10 and the increased of TIMP4 are responsible for the depressed growth and invasion of tumor cells due to S100A4 silencing, are currently being investigated in our laboratory.

Additionally, CDH11, type 2, OB-cadherin also showed much lower expression after S100A4 silencing. CDH11 is an integral membrane protein and mediates cell adhesion in a calcium-dependant manner (34). Overexpression of this molecule is strongly associated with tumor metastasis and recurrence in pleomorphic adenomas (35), breast cancer (36,37) and prostate cancer (38). In prostate cancer, it has been suggested that downregulation of CDH11 leads to inhibition of tumor cell migration *in vivo* and *in vitro* (38). CDH11 exhibited lower expression after S100A4 silencing in colorectal cancer cells, suggesting that it may correlate with S100A4. Further study of this hypothesis is under way.

In conclusion, our present study is the first to show that knockdown of S100A4 using RNA interference could effectively inhibit proliferation, invasion and metastasis in SW620 colorectal cancer cells. The function of S100A4 as an oncoprotein may be associated with several important molecules involved in growth, invasion and metastasis of cancer cells. These results further indicate that S100A4 may serve as a potential target for the development of therapies for colorectal cancer, although additional studies *in vivo* are necessary.

## Figures and Tables

**Table I:** Genes dysregulated by >2-fold after S100A4 silencing in SW620 cells identified by human tumor metastasis-related gene mRNA microarray analyses.

Abbreviations	mRNA ratio^a^	GenBank ID	Gene description	Character and function
TIMP4	2.18	7079	Tissue inhibitors of metalloproteinase 4	Inhibitors of matrix metalloproteinases
CDH11	0.39	1009	Cadherin 11, type 2, OB-cadherin	Integral membrane proteins that mediate calcium-dependent cell-cell adhesion
MMP9	0.46	4318	Matrix metallo-peptidase 9	Involved in the breakdown of the extra-cellular matrix in normal physiological processes as well as in disease processes
MMP10	0.40	4319	Matrix metallo-peptidase 10	Involved in the breakdown of the extra-cellular matrix in normal physiological processes as well as in disease processes

a mRNA ratio represents the abundance ratio between SW620-siRNA and SW620-siControl.
